# On the Influence of Ultimate Number of Cycles on Lifetime Prediction for Compression Springs Manufactured from VDSiCr Class Spring Wire

**DOI:** 10.3390/ma13143222

**Published:** 2020-07-20

**Authors:** Max Benedikt Geilen, Marcus Klein, Matthias Oechsner

**Affiliations:** Centre for Engineering Materials (MPA-IfW), TU Darmstadt, Grafenstrasse 2, 64283 Darmstadt, Hessia, Germany; m.klein@mpa-ifw.tu-darmstadt.de (M.K.); oechsner@mpa-ifw.tu-darmstadt.de (M.O.)

**Keywords:** VDSiCr, SWOSC-V, spring steel, fatigue, compressions springs, VHCF, FKM guideline

## Abstract

For the generation of fatigue curves by means of fatigue tests, an ultimate number of cycles must be chosen. This ultimate number of cycles also limits the permissible range of the fatigue curve for the design of components. This introduces extremely high costs for testing components that are to be used in the Very High Cycle Fatigue regime. In this paper, we examine the influence of the ultimate number of cycles of fatigue tests on lifetime prediction for compression springs manufactured from VDSiCr class spring wire. For this purpose, we propose a new kind of experiment, the Artificial Censoring Experiment (ACE). We show that ACEs may be used to permissibly extrapolate the results of fatigue tests on compression springs by ensuring that a batch-specific minimum ultimate number of cycles has been exceeded in testing. If the minimum ultimate number of cycles has not been exceeded, extrapolation is inadmissible. Extrapolated results may be highly non-conservative, especially for models assuming a pronounced fatigue limit.

## 1. Introduction

A standard for the minimum lifetime of compression springs under cyclic loading is given in EN 13906-1 [1]. This standard is a great help for design engineers because it allows a safe design at a reasonable design cost and a reasonable utilization of the materials used. However, the potential for lightweight design is not fully exploited. In addition, EN 13906-1 [1] is only applicable for up to 10${}^{7}$ cycles while valve springs are loaded for up to 10${}^{9}$ cycles in many applications, for example in automotive engines.

A new guideline for springs and spring elements [2] is currently being developed in a cooperation between Forschungskuratorium Maschinenbau (FKM), a German research association, the Technical University of Darmstadt and the Technical University of Ilmenau. Once published, it will allow designing for higher numbers of cycles as well as a better exploitation of lightweight potential. The design process using this new FKM guideline ‘Analytical Strength Assessment of Springs and Spring Elements’ will include specifying a parameter described as the level of trust in the data used to build the underlying model. In practice, this factor incorporates the influence of a given spring producer’s production process into the fatigue design rules of the guideline. This is implemented by raising or lowering the fatigue curve to make its predictions slightly conservative, but not too conservative for compression springs produced in the producer’s given production process.

This factor should be calibrated for a given production process using fatigue data, ideally fatigue curves. In this paper, we discuss the influence of the ultimate number of cycles on the resulting fatigue curves for compression springs manufactured from VDSiCr class valve spring wire (VDSiCr). Once tests up to 10${}^{7}$ cycles produce conservative results for lifetime predictions up to 10${}^{9}$ cycles, fatigue tests conducted only up to 10${}^{7}$ cycles may be safely used to calibrate the factor described above. This is relevant for spring manufacturers because testing springs with wire diameters of 1 to 3 mm up to 10${}^{9}$ cycles comes with a year of testing time and a six figure Euro price tag. To the author’s knowledge, there is no investigation of springs with larger diameters exceeding 10${}^{7}$ loading cycles.

**Figure 1:**
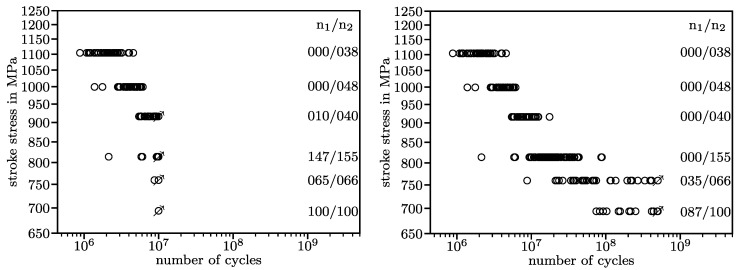
Results of a fatigue test up to 10${}^{7}$ cycles (**left**) and after 5·10${}^{8}$ cycles (**right**), data from [3].

As a primer to the question why testing up to 10${}^{7}$, which is common practice, may not be sufficient, see Figure 1. On the left side, a fatigue test that was interrupted after 10${}^{7}$ cycles is presented. For each horizon, the number of run-outs, n${}_{1}$, and the total number of specimens tested, n${}_{2}$, are given. On the right side, the result of the same fatigue test continued to 5·10${}^{8}$ cycles is presented. Looking at the diagram on the left, an engineer expecting a pronounced fatigue strength could conclude that at the lowest load level, the springs tested have an infinite lifetime. Confronted with the diagram on the right, we know that this conclusion is wrong.

In Section 2, the statistical model used in this investigation is introduced. The numerical implementation of an algorithm that fits a model to the data under investigation is described in Section 3. An extension to this implementation allowing an Artificial Censoring Experiment (ACE) is described there as well. In the ACE, a numerical thought experiment is conducted where we wonder what lifetime predictions we would have obtained if we would have conducted the fatigue experiment with a lower ultimate number of cycles than we actually did. This thought experiment may answer the question whether the ultimate number of cycles we actually used in our experiments influences the predicted lifetime, i.e., if our lifetime prediction is admissible. Fatigue results of compression springs manufactured from VDSiCr and the setup for our numerical experiments are presented in Section 4. The results are presented in Section 5. Possible extensions to other batches of compression springs manufactured from VDSiCr are discussed in Section 6. The whole investigation and its key results are summarized in Section 7.

## 2. Utilized Statistical Model for Fatigue Events

Fatigue results are customarily plotted with logarithmic scaling in both axes, Figure 1. In this coordinate system, fatigue curves of compression springs have historically been constructed as bilinear curves descending linearly in the High Cycle Fatigue regime and running horizontally in the Very High Cycle Fatigue regime. The transition was assumed to be somewhere between 10${}^{6}$ and 10${}^{7}$ cycles. This model is based on the assumption a pronounced fatigue strength in this range. Fatigue failures of components cyclically loaded in the gigacycle regime and fatigue tests on material specimens in the 1980s and 1990s [4] have challenged this assumption. Inspired by this challenge, researchers conducted fatigue experiments beyond 10${}^{7}$ cycles [5]. The results of these fatigue experiments disprove the assumption of a pronounced fatigue strength around 10${}^{6}$ or 10${}^{7}$ cycles for compression springs. This finding has been repeated in a multitude of tests on material specimens [6,7,8,9,10,11,12,13,14] and compression springs [3,15,16].

**Figure 2:**
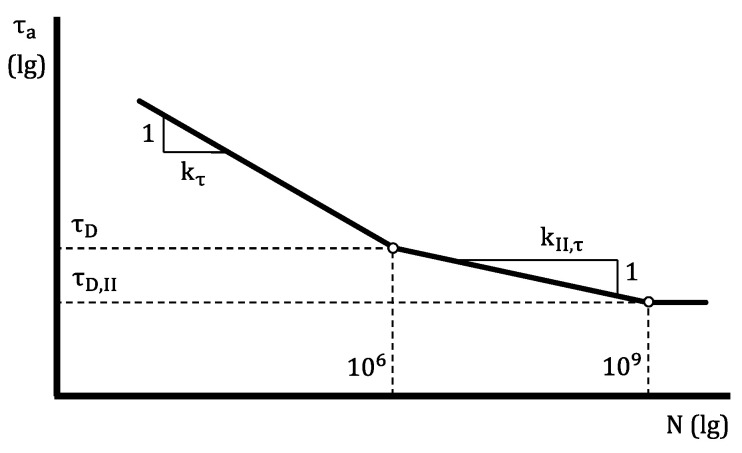
Model of the fatigue curve employed in the upcoming Forschungskuratorium Maschinenbau (FKM) guideline for springs and spring elements with stress amplitude $\tau_{a}$, number of cycles N stress amplitudes at kink points $\tau_{D}$ and $\tau_{D,\mathrm{II}}$ as well as slopes $k_{\tau }$ and $k_{\mathrm{II},\tau }$, modified from [2].

The model that is used by the upcoming FKM guideline for springs and spring elements [2] utilizes a trilinear fatigue curve, Figure 2. The fatigue curve kinks a first time at 10${}^{6}$ cycles slowing down its descent and a second time at 10${}^{9}$ cycles, running horizontally from there on. This is based on the assumption of a pronounced fatigue strength after 10${}^{9}$ cycles, which has not been proven to be false.

However, there is also no experimental evidence available that supports the assumption of a pronounced fatigue strength beyond 10${}^{9}$ cycles. Therefore, in this work the more conservative approach of a bilinear fatigue curve is used. The kinking of the fatigue curve occurs at different stroke stresses and different numbers of cycles depending on the properties of the batch of springs under investigation as well as the testing setup. The model used incorporates this through a variable kink point.

Fatigue events in metallic materials usually occur following a log-normal distribution [17,18,19]. Experiments on material specimens [20] as well as compression springs [16] have shown that this is not the case for VDSiCr. A key reason for this is the presence of multiple competing failure mechanisms. Problems arising from this have been addressed in literature [3,21,22,23,24,25,26,27,28], however consensus over a suitable solution has not yet been reached. In this investigation, a log-normal distribution in the direction of the number of cycles is assumed above and below the kink point. As evident in Figure 1, variance is much higher in lower load levels. Two independent variances are used above and below the kink point.

## 3. Numerical Implementation

### 3.1. Mathematical Description of the Model

**Figure 3:**
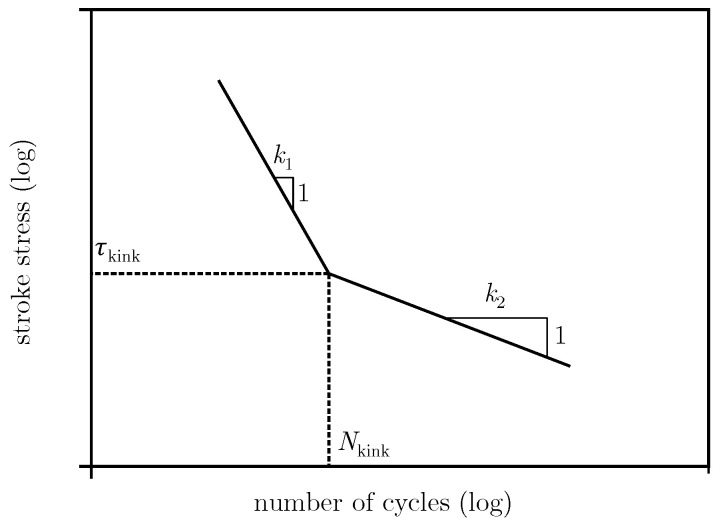
Parameters used in the numerical implementation of the model.

The model used to describe the fatigue behavior is described in Section 2. To implement the model numerically, a mathematical description is necessary. The curve describing a survival probability of 50% is described by the stress in the kink point, $\tau_{\mathrm{kink}}$, and the number of cycles in the kink point, $N_{\mathrm{kink}}$, as well as the slope of the curve above and below the kink point, $k_{1}$ and $k_{2}$. These parameters are displayed in Figure 3.

We now consider the probability distribution at the load level of the kink point, $\tau_{\mathrm{kink}}$. To make calculations easier, we use the logarithmic notation $N_{\log,\mathrm{kink}}=\log N_{\mathrm{kink}}$ and $\tau_{\log,\mathrm{kink}}=\log\tau_{\mathrm{kink}}$. For the time being, we assume a single standard deviation SD. Following the very definition of a 50%-survival-line, the logarithmic expectation value, $\mu_{\log}=\log\mu$, is mathematically described as:

(1) $$\mu_{\log}=N_{\log,\mathrm{kink}}$$

Based on the logarithmic expectation value, $\mu_{\log}$, for any logarithmic number of cycles, $N_{\log}$, the probability density function *f* may be computed.

(2) $$f\left(N_{\log}|\mu_{\log},SD\right)=\frac{1}{\sqrt{2\pi SD^{2}}}\exp\left(-\frac{{\left(N_{\log}-\mu_{\log}\right)}^{2}}{2\;SD^{2}}\right)$$

The integral of the density function *f* between two logarithmic numbers of cycles, $N_{\log,1}$ and $N_{\log,2}$, at a given load level $\tau_{\log}$ describes the probability that a specimen loaded at $\tau_{\log}$ will fail between $N_{\log,1}$ and $N_{\log,2}$ if loaded cyclically at $\tau_{\log}$. The integral from negative infinity to a logarithmic number of cycles $N_{\log}$ at $\tau_{\log}$ describes the probability that a given specimen will fail before or at $N_{\log}$. A negative value for the number of cycles is justified since we are working in a logarithmic system. In engineering terms, minus infinity in a logarithmic system translates to zero in a linear system. This integral is called the cumulative distribution function *F*. It may be calculated using the error function erf.

(3) $$F\left(N_{\log}|\mu_{\log},SD\right)=\frac{1}{2}\left(1+\mathrm{erf}\left(-\frac{{\left(N_{\log}-\mu_{\log}\right)}^{2}}{\sqrt{2SD^{2}}}\right)\right)$$

The survival function, *S*, describes the probability that a given specimen will not have failed at a given number of cycles.

(4) $$S\left(N_{\log}|\mu_{\log},SD\right)=1-F\left(N_{\log}|\mu_{\log},SD\right)=\frac{1}{2}\left(1-\mathrm{erf}\left(-\frac{{\left(N_{\log}-\mu_{\log}\right)}^{2}}{\sqrt{2SD^{2}}}\right)\right)$$

Each event *i* in a dataset (failure or run-out) is defined by a set of three parameters: the load level $\tau_{i}$, the cycle count $N_{i}$, and a flag variable $c_{i}$ that describes if the specimen failed. To include the event into our model, it is first transformed into a triplet in logarithmic space, ($N_{\log,i}$, $\tau_{\log,i}$, $c_{i}$). This triplet is constant throughout the whole process of finding the best model. During an ACE, the parameters $N_{\log,i}$ and $c_{i}$ may change.

The model described thus far only applies to fatigue data on the load level of the kink point, $\tau_{\mathrm{kink}}$. Since the events are not on that load level, they need to be moved there. This can be done by translating them parallel to the fatigue curve.

(5) $$N_{\log,\mathrm{kink},i}=\left\{\begin{array}{cc} N_{\log,i}+\left(\tau_{\log,i}-\tau_{\log,\mathrm{kink}}\right)\cdot k_{1}, & \mathrm{if}\;\tau_{i}>\tau_{\mathrm{kink}}\\ N_{\log,i}+\left(\tau_{\log,i}-\tau_{\log,\mathrm{kink}}\right)\cdot k_{2}, & \mathrm{if}\;\tau_{i}\leq \tau_{\mathrm{kink}} \end{array}\right.$$

Up to this point, a single standard deviation has been used. The model uses one standard deviation $SD_{1}$ for events above the kink point and another standard deviation $SD_{2}$ for events below the kink point. To this end, the probability density function *f*, the cumulative distribution function *F* and the survival function *S* are adjusted.

(6) $$f\left(N_{\log}|\mu_{\log},SD_{1},SD_{2},\tau \right)=\left\{\begin{array}{cc} \frac{1}{\sqrt{2\pi SD_{1}^{2}}}\exp\left(-\frac{{\left(N_{\log}-\mu_{\log}\right)}^{2}}{2\;SD_{1}^{2}}\right), & \mathrm{if}\;\tau >\tau_{\mathrm{kink}}\\ \frac{1}{\sqrt{2\pi SD_{2}^{2}}}\exp\left(-\frac{{\left(N_{\log}-\mu_{\log}\right)}^{2}}{2\;SD_{2}^{2}}\right), & \mathrm{if}\;\tau \leq \tau_{\mathrm{kink}} \end{array}\right.$$

(7) $$F\left(N_{\log}|\mu_{\log},SD_{1},SD_{2},\tau \right)=\left\{\begin{array}{cc} \frac{1}{2}\left(1+\mathrm{erf}\left(-\frac{{\left(N_{\log}-\mu_{\log}\right)}^{2}}{\sqrt{2SD_{1}^{2}}}\right)\right), & \mathrm{if}\;\tau >\tau_{\mathrm{kink}}\\ \frac{1}{2}\left(1+\mathrm{erf}\left(-\frac{{\left(N_{\log}-\mu_{\log}\right)}^{2}}{\sqrt{2SD_{2}^{2}}}\right)\right), & \mathrm{if}\;\tau \leq \tau_{\mathrm{kink}} \end{array}\right.$$

(8) $$S\left(N_{\log}|\mu_{\log},SD_{1},SD_{2},\tau \right)=\left\{\begin{array}{cc} \frac{1}{2}\left(1-\mathrm{erf}\left(-\frac{{\left(N_{\log}-\mu_{\log}\right)}^{2}}{\sqrt{2SD_{1}^{2}}}\right)\right), & \mathrm{if}\;\tau >\tau_{\mathrm{kink}}\\ \frac{1}{2}\left(1-\mathrm{erf}\left(-\frac{{\left(N_{\log}-\mu_{\log}\right)}^{2}}{\sqrt{2SD_{2}^{2}}}\right)\right), & \mathrm{if}\;\tau \leq \tau_{\mathrm{kink}} \end{array}\right.$$

### 3.2. Maximum Likelihood Fit

To find the model that describes the observed events best, a maximum likelihood estimation is conducted. This means that we vary the parameters used in the model above to find the one model that has the highest likelihood *L* of producing the underlying test results. The likelihoods of all events $L_{i}$ are merged multiplicatively, just like probabilities. To be able to process all observed events $n_{2}$, they are split into one set containing all run-outs $n_{1}$ and one set containing all fracture events $n_{0}$

(9) $$L=\prod_{i}^{n_{2}}L_{i}=\prod_{i}^{n_{1}}L_{i,\mathrm{run}-\mathrm{out}}\cdot \prod_{i}^{n_{0}}L_{i,\mathrm{fracture}}$$

For fracture events, the likelihood $L_{i,\mathrm{fracture}}$ of them occurring is computed using the probability density function.

(10) $$L_{i,\mathrm{fracture}}\left(N_{\log,i},\tau_{i}|\mu_{\log},SD_{1},SD_{2}\right)=\left\{\begin{array}{cc} \frac{1}{\sqrt{2\pi SD_{1}^{2}}}\exp\left(-\frac{{\left(N_{\log,i}-\mu_{\log}\right)}^{2}}{2\;SD_{1}^{2}}\right), & \mathrm{if}\;\tau >\tau_{\mathrm{kink}}\\ \frac{1}{\sqrt{2\pi SD_{2}^{2}}}\exp\left(-\frac{{\left(N_{\log,i}-\mu_{\log}\right)}^{2}}{2\;SD_{2}^{2}}\right), & \mathrm{if}\;\tau \leq \tau_{\mathrm{kink}} \end{array}\right.$$

For run-outs, the likelihood $L_{i,\mathrm{run}-\mathrm{out}}$ is computed as the integral over the likelihoods of all fracture events not ruled out by the specimen surviving thus far. This is equal to the survival function.

(11) $$L_{i,\mathrm{run}-\mathrm{out}}\left(N_{\log,i},\tau_{i}|\mu_{\log},SD_{1},SD_{2}\right)=\left\{\begin{array}{cc} \frac{1}{2}\left(1-\mathrm{erf}\left(-\frac{{\left(N_{\log,i}-\mu_{\log}\right)}^{2}}{\sqrt{2SD_{1}^{2}}}\right)\right), & \mathrm{if}\;\tau >\tau_{\mathrm{kink}}\\ \frac{1}{2}\left(1-\mathrm{erf}\left(-\frac{{\left(N_{\log,i}-\mu_{\log}\right)}^{2}}{\sqrt{2SD_{2}^{2}}}\right)\right), & \mathrm{if}\;\tau \leq \tau_{\mathrm{kink}} \end{array}\right.$$

It seems counterintuitive that one likelihood is computed as a probability density and the other one as a probability. The approach described above is, strictly speaking, not correct because it is assumed that we know exactly at which (floating point) number of cycles the specimen broke. There is no way of knowing from the data presented in Section 4 at which number of cycles exactly the specimens broke. No matter how precisely the number of cycles to fracture is observed, there will always be some uncertainty, even if the error is less than a single cycle. Therefore, strictly speaking, the likelihood of fracture events must be computed following rules for doubly-censored data. This would also make the computations more consistent. In this paper, we use the approach assuming that we know exactly at which number of cycles fracture events occurred. The alternative likelihood $L_{i,\mathrm{fracture},\mathrm{alt}}$ may only be applied if an estimation of the error of the number of cycles until failure is available. The alternative likelihood function with the minimum and maximum number of cycles at failure $N_{\log,i,\min}$ and $N_{\log,i,\max}$ is:

(12) $$\begin{array}{ll} L_{i,\mathrm{fracture},\mathrm{alt}}(N_{\log,i,\min}, & N_{\log,i,\max},\tau_{i}|\mu_{\log},SD_{1},SD_{2})\\ & =\left\{\begin{array}{cc} \frac{1}{\sqrt{2\pi SD_{1}^{2}}}\int_{N_{\log,i,\min}}^{N_{\log,i,\max}}\exp\left(-\frac{{\left(N-\mu_{\log}\right)}^{2}}{2\;SD_{1}^{2}}\right)\;dN, & \mathrm{if}\;\tau >\tau_{\mathrm{kink}}\\ \frac{1}{\sqrt{2\pi SD_{2}^{2}}}\int_{N_{\log,i,\min}}^{N_{\log,i,\max}}\exp\left(-\frac{{\left(N-\mu_{\log}\right)}^{2}}{2\;SD_{2}^{2}}\right)\;dN, & \mathrm{if}\;\tau \leq \tau_{\mathrm{kink}} \end{array}\right.\\ & =\left\{\begin{array}{cc} \frac{1}{2}\;\mathrm{erf}\left(-\frac{{\left(N_{\log,i,\max}-\mu_{\log}\right)}^{2}}{\sqrt{2SD_{1}^{2}}}\right)-\frac{1}{2}\;\mathrm{erf}\left(-\frac{{\left(N_{\log,i,\min}-\mu_{\log}\right)}^{2}}{\sqrt{2SD_{1}^{2}}}\right), & \mathrm{if}\;\tau >\tau_{\mathrm{kink}}\\ \frac{1}{2}\;\mathrm{erf}\left(-\frac{{\left(N_{\log,i,\max}-\mu_{\log}\right)}^{2}}{\sqrt{2SD_{2}^{2}}}\right)-\frac{1}{2}\;\mathrm{erf}\left(-\frac{{\left(N_{\log,i,\min}-\mu_{\log}\right)}^{2}}{\sqrt{2SD_{2}^{2}}}\right), & \mathrm{if}\;\tau \leq \tau_{\mathrm{kink}} \end{array}\right. \end{array}$$

Now that the likelihood function can be computed, an appropriate optimization algorithm is necessary to find the global maximum. Optimization algorithms have a much easier time finding optima for sums compared to products. Therefore, we utilize the log-likelihood function $L_{\log}$, which is the natural logarithm of the likelihood function.

(13) $$L_{\log}=\ln\;L=\sum_{i}^{n_{2}}\ln\;L_{i}=\sum_{i}^{n_{1}}\ln\;L_{i,\mathrm{run}-\mathrm{out}}+\sum_{i}^{n_{0}}\ln\;L_{i,\mathrm{fracture}}$$

**Figure 4:**
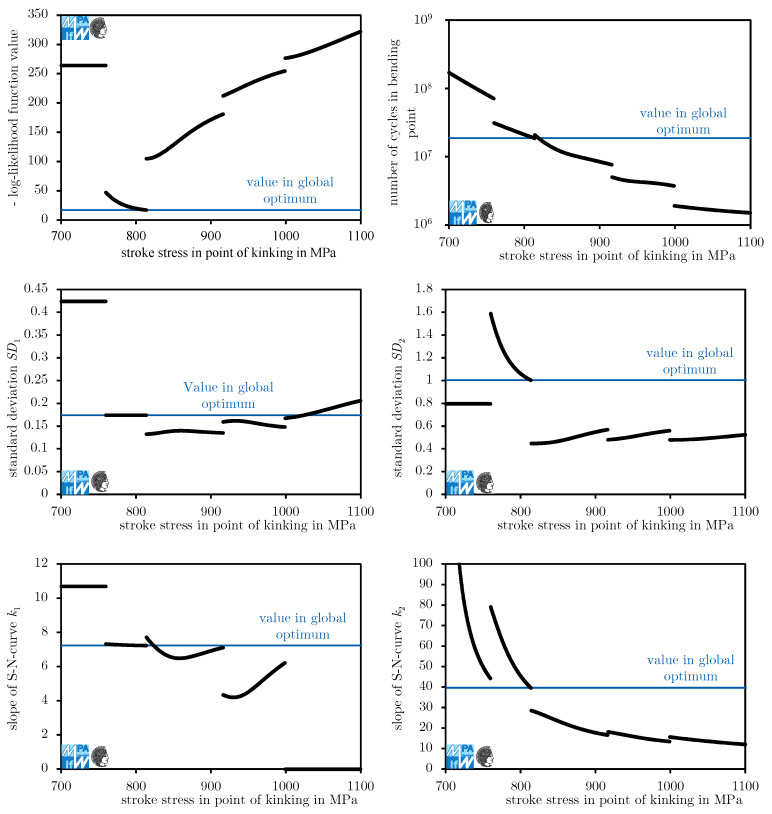
Likelihood function values and optimal parameters computed by optimizing maximum likelihood functions for fixed stresses (1000 points) and all other parameters optimized at the point of bending, $\tau_{\mathrm{kink}}$.

The log-likelihood function shows singularities around the load levels where tests were conducted. In Figure 4, the results of optimizations keeping the stress in the kink point $\tau_{\mathrm{kink}}$ constant and varying all other parameters are shown. The fatigue data presented in Figure 1 was used. The singularities can be pinpointed through jumps in all values. These singularities make it hard for local optimization algorithms to find the global optimum. Therefore, in this investigation, the search for the global maximum is split in two steps.

In the first step, the load stress in the kink point is varied in a brute force global search, varying all other parameters in a local search in each step using the SciPy implementation [29] of the L-BFGS-B algorithm [30,31]. L-BFGS-B is used because it solves quickly and is able to work with boundaries, which is important because without boundaries, the solver would diverge. For each iteration, the optimization results from the last iteration are used as a starting vector to save computing time.

In the second step, all parameters including the load stress in the kink point are varied in a local search using the SciPy implementation [29,32] of the downhill simplex method [33]. In the second step, the parameters of the result with the highest likelihood function value are used as a starting vector. Here, the downhill simplex method is used because it reliably converges to the local optimum. It converges slower than other algorithms and cannot handle boundaries, but that does not matter here because at the start of the search, we are close to the optimum already. The downhill simplex method alone may yield results that do not make sense from an engineering standpoint for the data used here—e.g., $k_{2}$ being lower than $k_{1}$. In addition, the algorithm might predict points of kinking at number of cycles higher than the ultimate number of cycles. This is counterintuitive and there is no evidence that such predictions can be reasonably justified. To eliminate these two phenomena, the likelihood function was expanded by a penalty term $L_{\mathrm{penalty}}$. The penalty term is computed using the Heaviside step function $H\left(x\right)=\frac{d}{dx}\;\max\left(x,0\right)$ and the user-defined penalty parameters $P_{k}$ and $P_{N}$.

(14) $$L_{\log,\mathrm{numeric}}=L_{\log}+L_{\mathrm{penalty}}=\sum_{i}^{n_{1}}\ln\;L_{i,\mathrm{run}-\mathrm{out}}+\sum_{i}^{n_{0}}\ln\;L_{i,\mathrm{fracture}}+L_{\mathrm{penalty}}$$

(15) $$L_{\mathrm{penalty}}=P_{k}\cdot H\left(k_{2}-k_{1}\right)\cdot {\left(k_{2}-k_{1}\right)}^{2}+P_{N}\cdot H\left(N_{\log,\mathrm{kink}}-\max\;N_{\log,i}\right)\cdot {\left(N_{\log,\mathrm{kink}}-\max\;N_{\log,i}\right)}^{2}$$

For this investigation, the penalty parameter $P_{N}$ is set to zero to avoid any corruption of the results by the approach that fatigue curves should not be extrapolated. The penalty parameter for the slopes of the fatigue curves is used throughout the whole paper, $P_{k}=10^{9}$.

The huge variation in parameters should be noted. Especially different slopes $k_{2}$ of fatigue curves below the kink point indicate that if fatigue curves are used for the extrapolation of results, predictions vary widely depending on the evaluation process. This is another reason not to extrapolate fatigue curves, besides the fact that the slope of the fatigue curve may increase toward higher numbers of cycles.

### 3.3. Variation of Ultimate Number of Cycles

In Section 3.1 and Section 3.2, a model to describe the fatigue curve for a given set of observed events was presented. In this section, an algorithm that produces data which helps in the assessment of the influence of the ultimate number of cycles on the resulting fitted model is described. The algorithm receives a dataset of observed events (triplets of data), which would traditionally be used to fit a model on.

First, a model is generated using the algorithm described in Section 3.2 based on the dataset without any artificial censoring. This is the reference model for the investigation. Ideally, if we decrease the ultimate number of cycles, the model fitted to the artificially censored dataset is the same as the model obtained without any artificial censoring. Of course, we do not expect that to be the case. To quantitatively capture the difference between the models, we compare the lifetime prediction for the 50% quantile of both models at different load levels (between the minimum and maximum load tested).

An array of artificially censored sets of observed events is generated. Each one is generated for a prescribed ultimate number of cycles in the following way: Starting with the original dataset, we consider each event (failure or run-out). If the number of cycles of the event is below the prescribed ultimate number of cycles, the event is left untouched. Otherwise, the events number of cycles is set to the prescribed ultimate number of cycles and the event is considered a run-out, regardless whether it is a failure or a run-out in the original dataset.

For each artificially censored fatigue test in the array created this way, models are generated using the algorithm described in Section 3.2. Lifetime predictions for the 50% quantile are generated at the same load levels as for the reference model. The lifetime predictions are normalized over the results of corresponding lifetime predictions for the reference model.

For each load level for which relative lifetime predictions have been generated, a graph is generated that shows the deviation in the lifetime prediction over the number of cycles at which the model has been artificially censored. A value lower than one means the result for the censored data is more conservative, a value higher than one means it is less conservative than the model generated with the original dataset.

## 4. Experimental Setup

Several datasets on the fatigue behavior of compression springs manufactured from VDSiCr are available. For ultimate numbers of cycles beyond 10${}^{7}$, fewer datasets are available. All available datasets were generated in a series of research projects at Technical University of Darmstadt [3,5,16,34].

**Figure 5:**
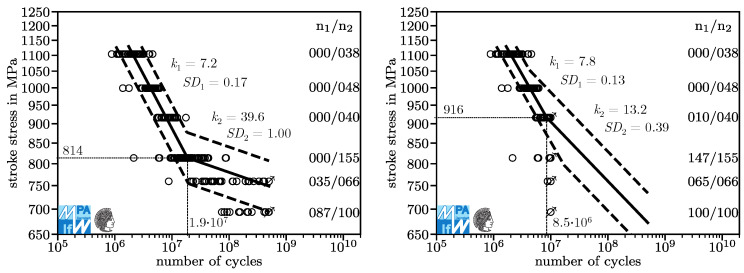
Fatigue curves with 10, 50, and 90% survival probability, generated from the original dataset (**left**) and from a dataset generated by artificial censoring at 10${}^{7}$ cycles (**right**), data from [3].

For this investigation, fatigue data from the most recent research project on the Very High Cycle Fatigue properties of compression springs, IGF 18576 N [3], are used. The data are presented in Figure 1. Fatigue curves with 10, 50, and 90% survival probability generated from the original dataset and from an artificially censored dataset are presented in Figure 5. The 50% fatigue curve on the left is used as a reference for lifetime prediction in the following investigation. It slices the results of all load levels at about the 50% quantile. This indicates it fits the data well.

Both curves look nearly the same up until 8.5·$10^{6}$ cycles. At over 8.5·$10^{6}$ cycles, the fatigue curve on the right predicts increasingly longer lifetimes than the one on the left, meaning that it is non-conservative and making it impermissible for fatigue design. The aim of the ACE is to identify this impermissibility with just the data on the right side available. For over 6·$10^{7}$, the right side model again makes more conservative lifetime predictions compared to the model on the left, which is dangerous since looking only at high and low load levels, leaving out the middle, one might assume the model on the right side is conservative.

The numerical experiment is split into two runs. In Run 1, the procedure is conducted using the model described in Section 3.1. In the analysis of Run 1, we observe some conservativeness due to an unrealistically fast descent of the fatigue curve below the kink point (unrealistically low $k_{2}$ of 13.2 for censoring at 10${}^{7}$, Figure 5). In Run 2, the exponent causing the unrealistically steep descent is fixed to the level provided in the new FKM guideline [2], $k_{2}=25$.

For each run, 100 datasets are generated by artificially censoring in logarithmically evenly spaced intervals between 10${}^{7}$ and 5·10${}^{8}$ cycles. For these datasets, lifetime predictions with a 50% chance of survival are created at stroke stresses of 700, 800, 900, 1000, and 1100 MPa. The lifetime predictions for the original dataset are given in Table 1. For higher load levels, lifetime predictions are similar. For lower load levels, Run 2 is more conservative.

## 5. Results

**Figure 6:**
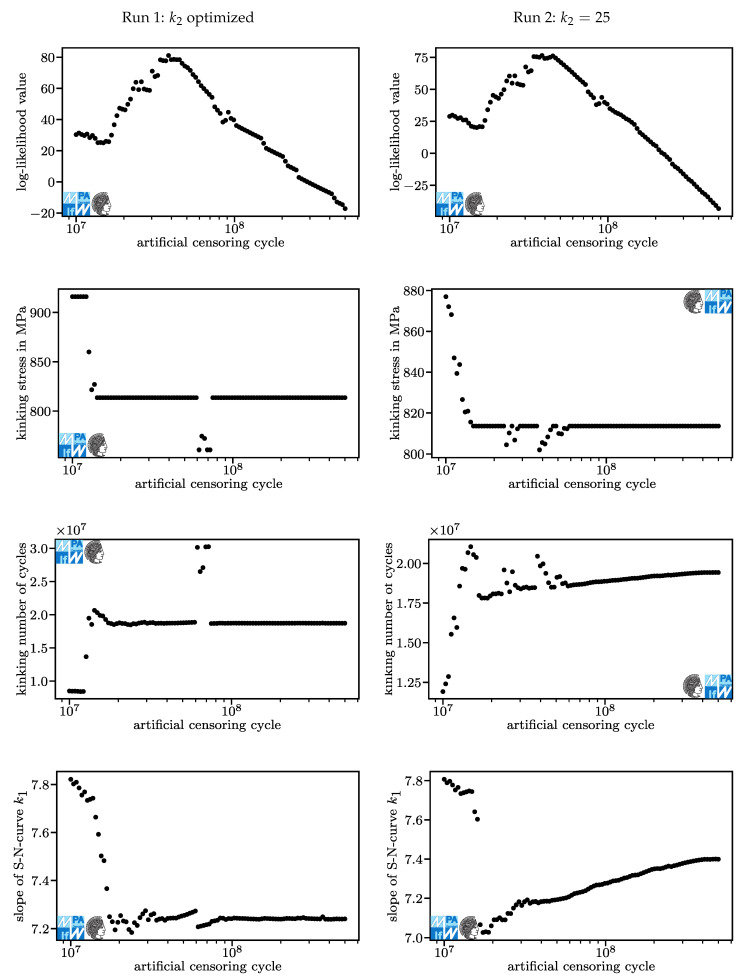
Results of optimization processes for Run 1 (**left**) and Run 2 (**right**) I.

**Figure 7:**
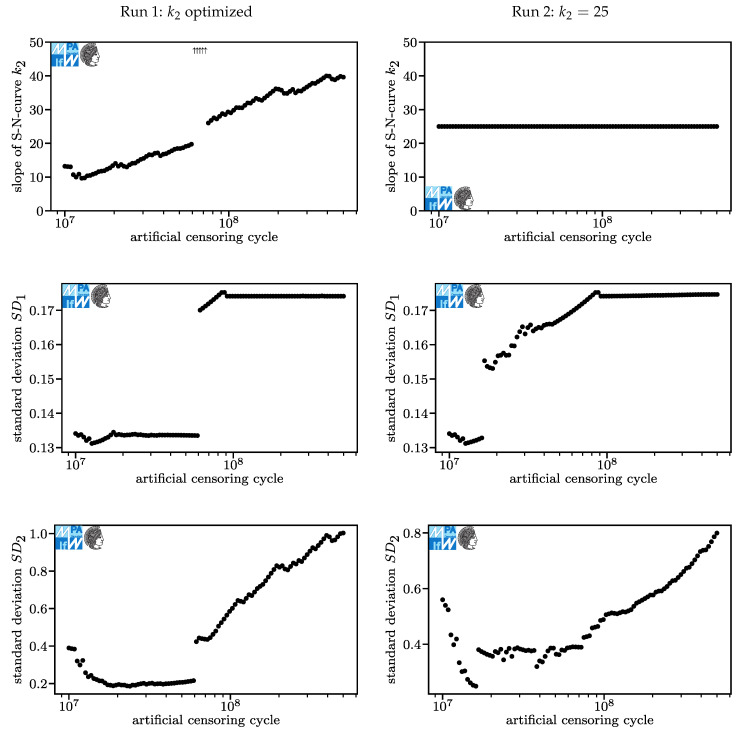
Results of optimization processes for Run 1 (**left**) and Run 2 (**right**) II.

The results of Run 1 and Run 2 are displayed in Figure 6 and Figure 7. In Figure 7 and the following Figures, arrows showing upwards and downwards mark data that is beyond the limits of the chart’s *y*-axis. Results for Run 1 are displayed on the left, results for Run 2 on the right. For each diagram, one point refers to one optimized model.

The points at the same artificial censoring cycle in different diagrams of the same run refer to the same model. The log-likelihood value is higher for Run 1 because one additional parameter could be varied. The only case where the log-likelihood value of Run 1 would be equal to that of Run 2 is if for Run 1, $k_{2}=25$, which implies that both models are equal. For artificial censoring cycles over 4·10${}^{7}$, the likelihood function falls approximately linearly (log-lin). This is mostly caused by run-outs moving towards higher numbers of cycles in a mostly constant model and thereby reducing the computed residual likelihood of fracture $L_{i,\mathrm{run}-\mathrm{out}}$.

In Run 1, for most models, the stress and the number of cycles in the kink point are roughly equal. At about 6.5·10${}^{7}$ artificial censoring cycles, five models vary momentously in their kink point. This jump in parameters does not translate to the log-likelihood value. For artificial censoring cycles below 1.5·10${}^{7}$, the kink point differs significantly from that at higher artificial censoring cycles. For Run 1, the stress and the number of cycles in the kink point each form another plateau. For Run 2, the stress in the kink point rises in an approximately (log-lin) linear fashion as the artificial censoring cycle reduces to 10${}^{7}$ while the number of cycles in the kink point falls after a swift rise. These differences are systematic biases, not random errors. This is a first warning sign not to decrease the ultimate number of cycles too far.

In both runs, the exponent $k_{1}$ for the slope only differs slightly. The reason for this is that $k_{1}$ helps fit the fatigue curve above the kink point, where the observed events are not affected by the artificial censoring. Its major changes occur where the kink point changes. This is very understandable because as the kink point moves above or below load levels where events occur, the dataset fitted by $k_{1}$ changes.

For Run 1, $k_{2}$ rises with greater artificial censoring cycles. Higher exponents $k_{2}$ mean that the fatigue curve approaches a horizontal course, which implies a pronounced fatigue strength. At the given maximum of 40 it is not horizontal. Further research is necessary to properly evaluate the question of whether a pronounced fatigue strength for compression springs manufactured from VDSiCr beyond 10${}^{9}$ cycles exists. For design purposes, one should conservatively assume it does not exist. For artificial censoring cycles around 6.5·10${}^{7}$ cycles, very high exponents $k_{2}$ occur. For these models, lifetime predictions may be highly non-conservative and therefore must me treated with utmost care. For Run 2, $k_{2}$ is constant.

Standard deviation above the kink point, $SD_{1}$, varies between 0.13 and 0.18 for both runs. For Run 1, it establishes two plateaus with a jump at an artificial censoring cycle of around 6.5·10${}^{7}$ cycles. For Run 2, a jump occurs earlier and a range of (log-lin) approximately linear growth occurs additionally to two plateaus at the same levels as the plateaus in Run 1.

Standard deviation below the kink point, $SD_{2}$, varies widely. One reason for its growth is that failures at the lowest load levels do not follow a log-normal distribution and have a higher scatter range than failures at medium load levels. If the data are artificially censored at a relatively low number of cycles, the lowest load levels are only factored in as run-outs. Run-outs occurring at cycle counts below the expectation value do not contribute positively to a greater standard deviation.

**Figure 8:**
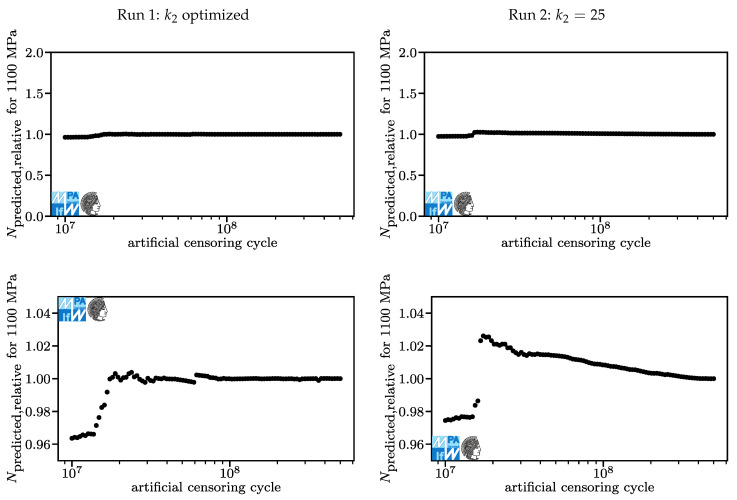
Lifetime predictions for Run 1 (**left**) and Run 2 (**right**), overview and detail for stroke stresses of 1100 MPa.

Lifetime predictions for the 50% quantile derived from the models in Figure 6 and Figure 7 for a stroke stress of 1100 MPa are displayed in Figure 8. Again, the results derived in Run 1 are shown on the left, the results from Run 2 are shown on the right. The upper graphs show the predictions of the models derived from censored data normalized over the predictions of the model with the original dataset, $N_{\mathrm{predicted},\mathrm{relative}}$. Here, an ideal value exists and it is 1.0. The last point has per definition the value 1.0. A value of 2.0 means that the model based on a censored dataset predicts a lifetime of twice as many cycles as the model based on the original dataset. This is bad because it is non-conservative. A value of 0.5 means that the model based on a censored dataset predicts a lifetime of half as many cycles as the model based on the original dataset. This is less bad because it is conservative. However, it is not good either since it prevents engineers from fully exploiting lightweight potential. The lower charts show the same data as the upper charts with different limits. Kindly note that a value of 1.0 on the left side corresponds to a different number of cycles than a value of 1.0 on the right side, Table 1. For Figure 8, only the lower part is relevant. Lifetime predictions differ by less than five percent. This is acceptable.

**Figure 9:**
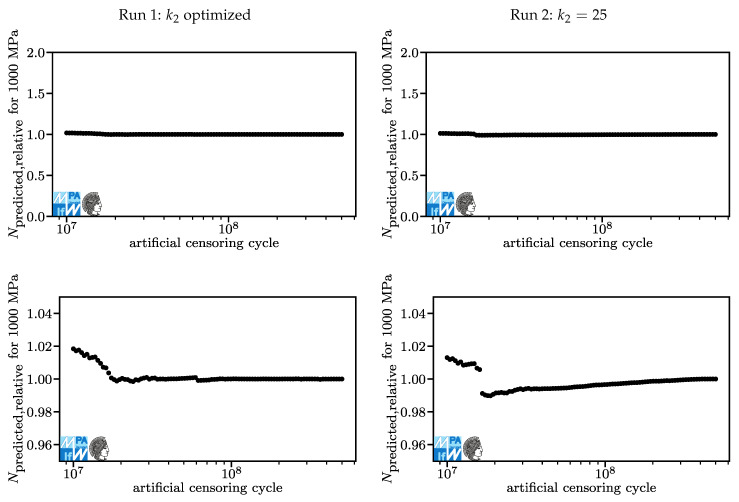
Lifetime predictions for Run 1 (**left**) and Run 2 (**right**), overview and detail for stroke stresses of 1000 MPa.

In Figure 9, the relative lifetime predictions for a stroke stress of 1000 MPa are displayed. Here, lifetime predictions also differ by less than five percent. The fact that up until this point, only small deviations occurred is of little surprise, considering that the lifetime prediction of the model fitted to the original dataset is 4.2·10${}^{6}$ for both runs, which is below the lowest censoring number of cycles under investigation. The highest deviations for Run 1 occur at artificial censoring cycles under 1.5·10${}^{7}$, which is where the kink point is at another plateau than without censoring. Here, fewer events are used for fitting the part of the fatigue curve above the kink point. The difference in the underlying dataset explains the difference in this part of the model. There is no obvious answer to the question of which prediction is better here. For Run 2, at artificial censoring cycles below 1.5·10${}^{7}$, the same effect produces a similar behavior.

The greater differences for higher artificial censoring cycles in Run 2 compared to Run 1 can be attributed to the algorithm having a hard time fitting the part of the fatigue curve below the kink point due to the fixed exponent $k_{2}$. This is understandable if one considers that the maximum likelihood method with a normal distribution is identical to least squares if no run-outs are present. Basically, a lot of measured error is created in the process of fitting the curve below the kink point because the curve just cannot fit well as the slope is fixed. Some of the error is transferred from the part of the model below the kink point to the part above the kink point. This can be seen in the rise in the number of cycles in the kink point, while the stress in the kink point is mostly constant, Figure 6. This does not occur for Run 1 because the transfer of error itself produces additional error overall.

**Figure 10:**
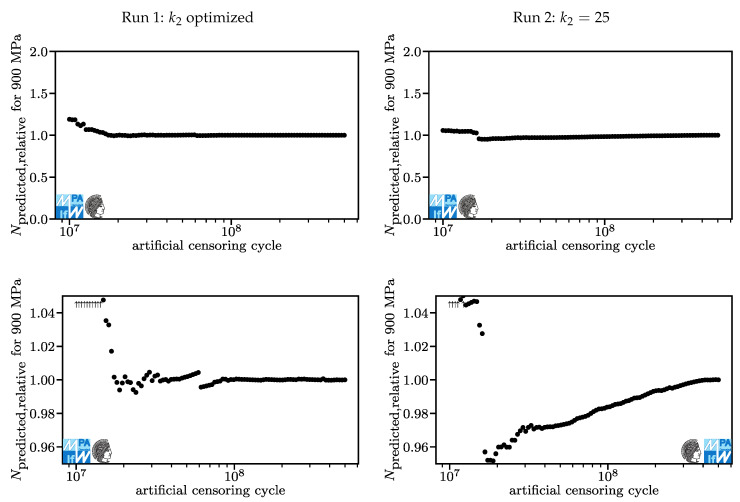
Lifetime predictions for Run 1 (**left**) and Run 2 (**right**), overview and detail for stroke stresses of 900 MPa.

In Figure 10, the relative lifetime predictions for a stroke stress of 900 MPa are displayed. For artificial censoring cycles over 2·10${}^{7}$, lifetime predictions are slightly conservative. Still being within five percent, this deviation is relatively small. For artificial censoring cycles below 2·10${}^{7}$, lifetime predictions become more non-conservative, peaking at 19% with artificial censoring at 10${}^{7}$ cycles. The lifetime prediction generated with the original dataset was 9.0·10${}^{6}$ for Run 1 and 9.2·10${}^{6}$ For Run 2. It is surprising to see momentous changes in lifetime prediction although all artificially censored tests still had ultimate numbers of cycles higher than the original lifetime prediction (although not higher than the lifetime prediction of the model based on the test with artificial censoring at 10${}^{7}$ cycles in Run 1).

In Run 1, a jump that corresponds to the jump in the model parameters occurs around 6.5·10${}^{7}$ cycles. This jump may also be visible is Figure 9 although this is less obvious and may be disputed by some viewers.

**Figure 11:**
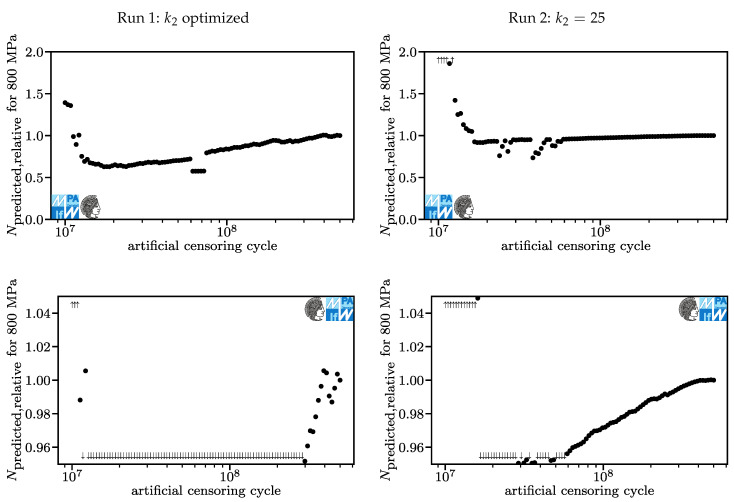
Lifetime predictions for Run 1 (**left**) and Run 2 (**right**), overview and detail for stroke stresses of 800 MPa.

In Figure 11, the relative lifetime predictions for a stroke stress of 800 MPa are displayed. The predicted lifetime of the model based on the original data is 3.7·10${}^{7}$ for Run 1 and 3.0·10${}^{7}$ for Run 2. This is where we expect results to differ momentously if fatigue curves truly should not be extrapolated. Unsurprisingly, predictions with artificial censoring cycles under 1.5·10${}^{7}$ are non-conservative, especially in Run 2, where lifetime is overestimated up to fourfold (up to 39% overestimation for run1).

Surprisingly, tests with artificial censoring cycles over 2·10${}^{7}$ are never non-conservative beyond five percent for both runs. For Run 1, the singularity around 6.5·10${}^{7}$ is more pronounced than in Figure 10, two jumps can be identified. For both runs, the relative lifetime prediction can be approximated well by a (log-lin) linear function. Run 2 shows better convergence behavior than Run 1. Kindly note that convergence behavior alone does not mean that a method is superior here because the curves converge to different values.

**Figure 12:**
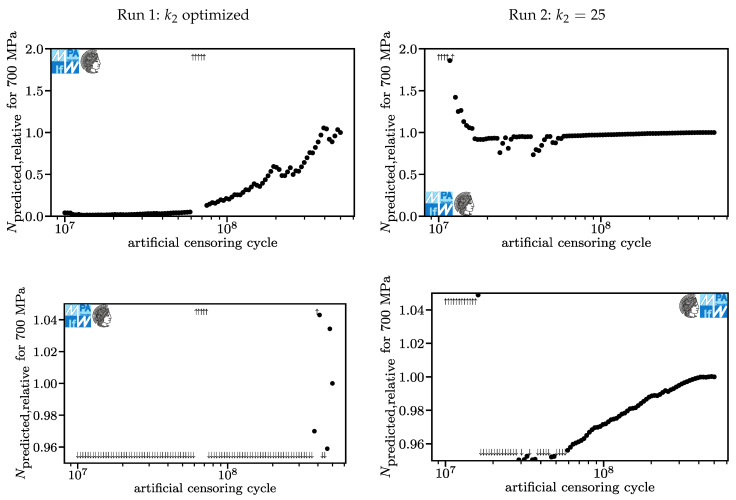
Lifetime predictions for Run 1 (**left**) and Run 2 (**right**), overview and detail for stroke stresses of 700 MPa.

In Figure 12, the relative lifetime predictions for a stroke stress of 700 MPa are displayed. The predicted lifetime of the model based on the original data is 7.3·10${}^{9}$ in Run 1 and 8.3·10${}^{8}$ in Run 2. Here, even the prediction of the model based on the original dataset is inadmissible for fatigue design. The assumption of a correct value of 1.0 appears not to be reasonable here. For Run 1, we see very conservative results for low artificial censoring cycles with relative lifetime prediction more or less continuously rising to 1.0 towards higher artificial censoring cycles. An exception to this is the range around 6.5·10${}^{7}$ cycles, where very non-conservative lifetime predictions are made. This is based on a very high exponent $k_{2}$ which corresponds to the false assumption of a fatigue strength somewhere around 10${}^{6}$ or 10${}^{7}$ cycles.

For Run 2, the charts are identical to the charts in Figure 11, except for the labels. This is caused by both load levels being below the kink point in all models and $k_{2}$ being constant.

## 6. Discussion

In Section 5, the results of an ACE for the batch of springs under investigation have been presented. In this section, we discuss which of these results may be generalized and used in the fatigue design of other batches of compression springs manufactured from VDSiCr.

The most alarming results of the investigation are very non-conservative results generated by tests running unil an ultimate number of cycles which is under 1.5·10${}^{7}$. Formulated positively, the results were safe to a certain degree when the ultimate number of cycles exceeded a minimum ultimate number of cycles, which for this batch was about 1.5·10${}^{7}$. We believe this to be a pattern having to do with the kinking of the fatigue curve and therefore being present in all batches of compression springs manufactured from VDSiCr. To our knowledge, there is no evidence supporting the idea that the kinking patterns of different batches of compression springs occur at the same number of cycles. Therefore, there is also no evidence supporting a fixed minimum ultimate number of cycles for all batches of compression springs manufactured from VDSiCr. This makes a simple recommendation like ultimate numbers of cycles of at least 2·10${}^{7}$ impossible at the current state of research.

Despite the lack of such a simple rule, the question of whether the ultimate number of cycles of a given fatigue test is greater or lower than the minimum ultimate number of cycles of the batch under investigation can still be examined. The examination is conducted by generating relative lifetime predictions for a variable exponent $k_{2}$ and for a fixed exponent $k_{2}=25$ in one ACE each and comparing the results for censoring cycles defined just below the ultimate number of cycles. Similar lifetime predictions for both ACEs at given censoring cycles indicate that the minimum ultimate number of cycles has been exceeded by the ultimate number of cycles and the results of the fatigue test may safely be used. Strongly deviating lifetime predictions at given censoring cycles indicate that this is not the case (or some other problem with one of the models is present, this is addressed below). Looking at Figure 11, this method would have told us that we chose the ultimate number of cycles too low if we would have tested for 1.5·10${}^{7}$ cycles.

Conducting ACEs with just one model may also help identify a minimum ultimate number of cycles that should be exceeded. Looking at Figure 6 and Figure 7, one recognizes huge gradients in different optimized parameters. If these pop up close to the ultimate number of cycles, design engineers should proceed with utmost care.

What is the advantage of the approach described above? Using this approach, we could have fitted a model to the results of an experiment with an ultimate number of cycles of 5·10${}^{7}$, correctly predicting that we do not introduce an unreasonable bias and saving 90 percent of testing time compared to the original setup. Testing up until around 6.5·10${}^{7}$, we would have correctly predicted that there is something wrong with the lifetime prediction at a stroke stress of 800 MPa because of the jump occurring just below our ultimate number of cycles. This analysis may even be conducted during testing, allowing an optimized ultimate number of cycles. Termination criteria must be defined before testing to prevent an inadmissibly subjective influence on the result of the fatigue test.

The results for the lowest load level deviated too much depending on the method to derive legitimate recommendations for the fatigue design of compression springs manufactured from VDSiCr beyond 10${}^{9}$ cycles. Here, we strongly recommend an ultimate number of cycles that is greater than any lifetime prediction made based on the test. Anything else would be especially imprudent because the fatigue curve may start falling quickly a second time at some number of cycles higher than 10${}^{9}$. Kindly note that according to the logic described above, the deviations are a stark warning sign that the ultimate number of cycles in testing is not high enough to make lifetime predictions at this level.

In literature, testing of compression springs with ultimate number of cycles of e.g., $10^{7}$ without additional analysis like an ACE is common practice. In the light of our findings, this practice should be evaluated critically by research institutions since results may be biased. The most obvious explanation for this bias is the mismatch between the model employed and the true fatigue behavior of the springs under investigation. This mismatch has been discussed in literature [3,21,22,23,24,25,26,27,28] but no consensus has been reached yet. In future investigations, ACEs may be used to compare the predictive power of different models without prior assumptions regarding the true fatigue behavior. The introduction of power analysis may help differentiate between non-conservative predictions due to a low number of failure events and bad predictions due to systematically excluding failure mechanisms by censoring events beyond the ultimate number of cycles.

## 7. Conclusions

In this paper, three algorithms were presented. The first algorithm fits a model to a set of observed events using the maximum likelihood method. The second algorithm creates artificially censored datasets by removing all information that was not present at a given number of cycles from an original dataset.

The third algorithm uses the second algorithm to create an array of sets with different artificial censoring cycles. It fits a model to each dataset and predicts lifetimes at different load levels. The lifetime predictions may be used for investigations regarding the influence of the ultimate number of cycles on the result of tests. This is a new kind of experiment, for which we propose the name Artificial Censoring Experiment (ACE).

In this paper, the fatigue behavior of compression springs manufactured from VDSiCr was investigated by means of the described ACE. The most important recommendations for engineers conducting fatigue design of such springs are:

- Without further investigation, to create a fatigue design for *n* cycles, a fatigue experiment must be conducted with an ultimate number of cycles of at least *n*.
- ACEs allow an extrapolation to a certain extent if a minimum ultimate number of cycles is exceeded.
- The minimum ultimate number of cycles may or may not depend on the batch of springs under investigation. For the batch under investigation in this paper, it was around 5·10${}^{7}$ cycles.
- If confronted with a very high computed exponent $k_{2}$, implying a pronounced fatigue strength, always conduct an ACE or use $k_{2}=25$. The computed very high exponent $k_{2}$ is probably caused by a too low ultimate number of cycles in the underlying fatigue test. Lifetime predictions for loads below the assumed pronounced fatigue strength may be wrong by multiple orders of magnitude.
- If designing for more than 10${}^{9}$ cycles, proceed with utmost care. Due to limited data in literature, we have no knowledge of the course of the fatigue curve beyond 10${}^{9}$ cycles. The ACEs conducted in this paper showed a dangerously high variance in predictions.

For researchers, the ACE offers an opportunity to research the influence of the ultimate number of cycles of other batches of compression springs manufactured from VDSiCr, potentially allowing a generalized rule for the extrapolation of fatigue data. Of course, this kind of research also should be conducted for other components and materials.

Further research should be conducted regarding probability distributions and potentials for lifetime predictions beyond 10${}^{9}$ cycles. All fatigue data available for compression springs with ultimate numbers of cycles over 10${}^{7}$ have been generated at a single institute making all datasets codependent. Therefore, additional data should be generated by other institutes.

In this paper, a basic ACE was proposed. More sophisticated experiments should be developed. For example an ACE setup allowing continuous interpretations in stress direction instead of interpretations at a very limited number of load levels would allow more objective investigations.

## Figures and Tables

**Table 1:** Lifetime prediction with 50% survival probability for models generated based on original dataset.

Load Level	700 MPa	800 MPa	900 MPa	1000 MPa	1100 MPa
prediction Run 1	7.3·10${}^{9}$	3.7·10${}^{7}$	9.0·10${}^{6}$	4.2·10${}^{6}$	2.1·10${}^{6}$
prediction Run 2	8.3·10${}^{8}$	3.0·10${}^{7}$	9.2·10${}^{6}$	4.2·10${}^{6}$	2.1·10${}^{6}$
